# Systematic review and pooled analysis of the impact of treatment-induced lymphopenia on survival of glioblastoma patients

**DOI:** 10.1186/s13014-023-02393-3

**Published:** 2024-03-14

**Authors:** Ali M. Saeed, Søren M. Bentzen, Haroon Ahmad, Lily Pham, Graeme F. Woodworth, Mark V. Mishra

**Affiliations:** 1 Department of Radiation Oncology, University of Maryland Greenebaum Comprehensive Cancer Center, University of Maryland School of Medicine, Baltimore, USA; 2 Maryland Proton Treatment Center, Baltimore, MD USA; 3 Department of Epidemiology and Public Health, Division of Biostatistics and Bioinformatics, University of Maryland School of Medicine, Baltimore, USA; 4 Department of Medical Oncology, University of Maryland Greenebaum Comprehensive Cancer Center, University of Maryland School of Medicine, Baltimore, USA; 5 Department of Neurosurgery, University of Maryland School of Medicine, Baltimore, MD USA

**Keywords:** Lymphopenia, Radiation therapy, Glioblastoma, Glioma, Proton beam therapy, Protons, Temozolomide

## Abstract

**Purpose/objective(s):**

Treatment related lymphopenia is a known toxicity for glioblastoma (GBM) patients and several single-institution studies have linked lymphopenia with poor survival outcomes. We performed a systematic review and pooled analysis to evaluate the association between lymphopenia and overall survival (OS) for GBM patients undergoing chemotherapy and radiation therapy (RT).

**Materials/methods:**

Following PRISMA guidelines, a systematic literature review of the MEDLINE database and abstracts from ASTRO, ASCO, and SNO annual meetings was conducted. A pooled analysis was performed using inverse variance-weighted random effects to generate a pooled estimate of the hazard ratio of association between lymphopenia and OS.

**Results:**

Ten of 104 identified studies met inclusion criteria, representing 1,718 patients. The lymphopenia cutoff value varied (400–1100 cells/uL) and as well as the timing of its onset. Studies were grouped as time-point (i.e., lymphopenia at approximately 2-months post-RT) or time-range (any lymphopenia occurrence from treatment-start to approximately 2-months post-RT. The mean overall pooled incidence of lymphopenia for all studies was 31.8%, and 11.8% vs. 39.9% for time-point vs. time-range studies, respectively. Lymphopenia was associated with increased risk of death, with a pooled HR of 1.78 (95% CI 1.46–2.17, P < 0.00001) for the time-point studies, and a pooled HR of 1.38 (95% CI 1.24–1.55, P < 0.00001) for the time-point studies. There was no significant heterogeneity between studies.

**Conclusion:**

These results strengthen observations from previous individual single-institution studies and better defines the magnitude of the association between lymphopenia with OS in GBM patients, highlighting lymphopenia as a poor prognostic factor.

## Introduction

Survival rates for glioblastoma remain poor despite advances in treatments that have iteratively extended survival times. The current standard of care involves maximal safe resection in combination with chemotherapy and radiation therapy (RT), followed by chemotherapy alone. Though these treatments have level I evidence for extending survival, they are associated with toxicities, some of which can be severe. An increasingly recognized treatment-related toxicity for various cancers including glioblastoma (GBM) is lymphopenia. The significance of this problem is highlighted by the well documented association between poorer survival outcomes and lymphopenia across various cancers, including GBM, esophageal, breast, cervical, lung, and pancreatic cancers [1–4].

The three main GBM treatments that contribute toward treatment-related lymphopenia are RT, chemotherapy, and corticosteroids – all of which are typically utilized during the course of GBM treatment, and independently contribute towards lymphopenia [4]. In addition, even prior to treatment GBM patients exhibit lymphopenia due to bone marrow sequestration of T cells [5]. The most widely used chemotherapy agent for GBM is temozolomide (TMZ) and it has myelosuppressive activity that may lead to lymphopenia [6, 7]. In the setting of GBM, RT may induce lymphopenia due to irradiation of circulating lymphocytes, which are among the most radiosensitive cell types [8, 9]. As modeled by Yovino et al., over the course of a typical GBM radiation therapy plan, about 99% of the circulating lymphocyte pool receives a lethal dose of radiation [9]. Combining these treatments (i.e., TMZ and RT) can lead to at least additive lymphocyte suppression. In the pre-TMZ-era, Hughes et al. demonstrated that RT-alone led to lymphopenia in about 24% of high-grade glioma patients [10]. A subsequent prospective observational study of high-grade glioma patients undergoing combination TMZ + RT found a much higher incidence of lymphopenia (40%) and lymphocytes remained suppressed for up to a year [10]. Moreover, patients with lymphopenia from Grossman et al.’s cohort had worse survival outcomes when compared with those that did not develop lymphopenia (median survival of 13.1 months vs. 19.7 months, respectively) [11].

Following this seminal publication, a number of studies have examined the association between lymphopenia and survival outcomes of GBM patients undergoing chemo-radiation therapy (CRT). In the present study we review and identify the available literature examining the association of treatment-related lymphopenia on the survival of GBM patients. In addition, we conducted a pooled analysis to better quantify and measure the magnitude of the association between treatment related lymphopenia and survival outcomes.

## Methods

### Literature search

This systematic review and pooled-analysis followed the PRISMA guidelines [12]. Primary clinical studies were identified by querying the PubMed MEDLINE database. The search was conducted using the following keywords: “lymphopenia”, “glioma OR glioblastoma”, “radiation OR radiotherapy”, with additional search employing MeSH terms “Lymphopenia[Mesh]”, “Glioma[Mesh]”, and “Radiotherapy[Mesh]”. Additionally, abstracts were also identified from the annual meetings of American Society for Radiation Oncology (ASTRO), American Society of Clinical Oncology (ASCO), and Society of Neuro-Oncology (SNO) using the keywords “lymphopenia”, “glioma OR glioblastoma”, “radiation OR radiotherapy”. Articles were last collected on September 2022. Inclusion criteria included the following: (1) retrospective or prospective clinical studies of human subjects (2) included high-grade glioma (HGG) patient, grade III or grade IV (where the majority of the entire cohort was HGG, and of the HGG cohort, majority were GBM/grade IV) (3) treatment involved combination chemotherapy and radiation therapy (4) reported lymphopenia outcomes (5) reported survival outcome (6) analyzed association between lymphopenia and survival. Exclusion criteria included the following: (1) low-grade glioma only patients (2) non-human studies (3) non-English language manuscripts or abstracts (4) patients treated with either chemotherapy alone/radiation therapy alone/surgery alone. In the case of manuscripts from the same institution covering overlapping inclusion times, only the most recent manuscript was included to prevent analysis of overlapping patient populations.

### Statistical analysis

The primary outcome of interest from the collected studies was a determination of the hazard ratio associated between the incidence of lymphopenia and overall survival (OS). To quantify this, a fixed-effect, inverse variance-weighted analysis of the logarithm of the hazard ratio (HR) of association between lymphopenia and OS was conducted using Review Manager 5.3. The pooled HR is reported with 95% CI, and p-values are 2-sided. A chi-square test for heterogeneity, the fraction of variance due to heterogeneity (I^2^), and a Z-test for overall effect were all estimated using RevMan.

## Results

**Fig. 1:**
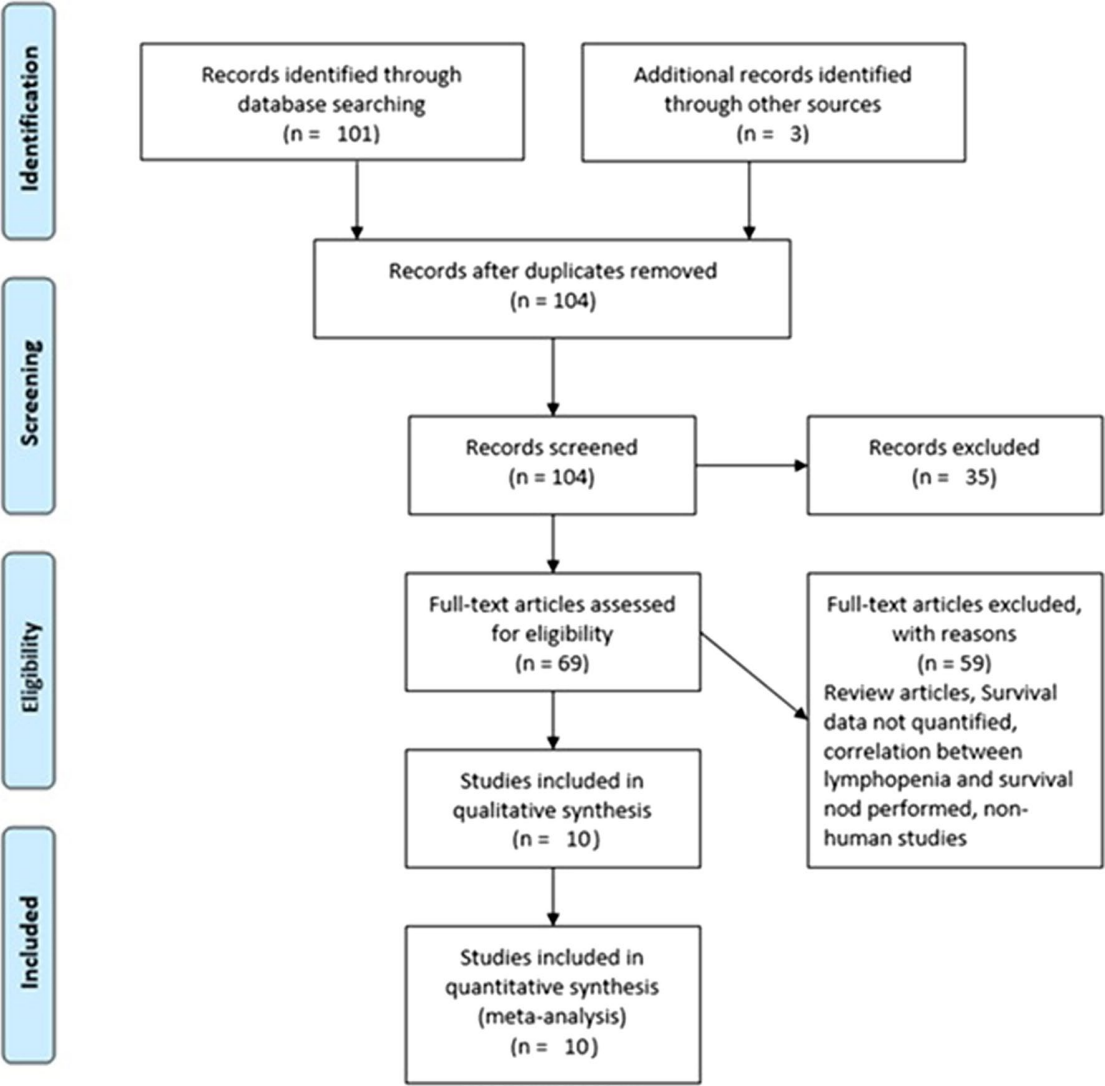
PRISMA literature review scheme

Based on the parameters delineated in the materials and Methods sections, after screening an initial 104 potential studies (Fig. 1), 10 studies met the inclusion criteria and were included for analysis (Table 1) [11, 13–20]. Briefly, these were studies including majority GBM patient cohorts undergoing combination chemotherapy and RT, with analysis of an association between lymphopenia and OS. Eight of the studies were single-institution retrospective series, one was a multi-center prospective observational study, and one was a single-institution phase II randomized control trial. Eight of the studies were exclusively GBM/grade 4 glioma cohorts, while one study (Grossman et al.) was entirely HGG (i.e., grade 3 and 4 with majority [85%] GBM) [21]. Another study included grade 2 gliomas (Ahn et al.), but HGG represented the majority of patients (66%), and GBM patients were the largest fraction of patients of the HGGs [18]. Collectively, the 10 included studies represent 1,718 unique patients.

**Table 1:** Characteristics of included studies

	Year	Author	Study	Sites	No. of patients	Median Age	% HGG	% Grade 4	Lymphopenia cutoff	Lymphopenia event definition	Time Classification	Incidence of lymphopenia	Median OS of lymphopenia group	Median Survival of non-lymphopenia group	p-value of OS difference
1	2011	Grossman	Prospective	Multicenter	96	57	100%	85%	< 200 CD4/cc	At 2-months after start of CRT	time-point	39.60%	13.1	19.7	0.002
2	2016	Rahman	Retrospective	Single	196	59	100%	100%	< 500 TLC/cc	Any time from start of CRT to 6 weeks after	time-range	47%	14.1	18.2	0.003
3	2017	Turkaj	Retrospective	Single	127	61	100%	100%	NR	Any time from start of CRT to 2.5 months after	time-range	NR	16.8	25.5	0.04
4	2018	Song	Retrospective	Single	96	61	100%	100%	< 400 TLC/cc	At 1-month after CRT completion	time-point	13.50%	8.4	18	0.041
5	2019	Byun	Retrospective	Single	336	58	100%	100%	< 500 TLC/cc	Any time from start of CRT to 3-months after	time-range	35.10%	18.2	22	0.028
									< 500 TLC/cc	At 3-months after start of CRT	time-point	NR	NR	NR	NR
6	2019	Hui	Retrospective	Single	319	57	100%	100%	< 500 TLC/cc	Any time from start of CRT to 3-months after	time-range	NR	NR	NR	NR
7	2019	Kim	Retrospective	Single	219	54	100%	100%	< 500 TLC/cc	At 1-month after CRT completion	time-point	2.90%	13	19.5	0.011
8	2019	Ye	Retrospective	Single	148	47	66.20%	43.90%	< 1,000 TLC/cc	Any time from start of CRT to end of CRT	time-range	46.60%	29	61.5	0.004
9	2020	Ahn	Retrospective	Single	97	59	100%	100%	< 1,000 TLC/cc	At 1-month after CRT completion	time-point	44%	14.5	21	0.017
10	2020	Mohan	Prospective	Single	84	53	100%	100%	< 500 TLC/cc	Any time from start of CRT to 1-month after	time-range	31%	20.8	25.1	0.46

cc = cubic centimeters; NR = not reported; TLC = Total lymphocyte count; RT = radiation therapy; CRT = chemo-radiotherapy

In reviewing the 10 primary studies, there was significant heterogeneity in defining lymphopenia, ranging from 200 to 1,000 total lymphocyte count per cubic centimeter. Similarly, there was not a defined time at which the incidence of lymphopenia was tabulated (e.g., during active CRT versus after CRT). Also, there was a key distinction in how lymphopenia in relation to time was reported. Five of the studies tabulated the incidence of lymphopenia defined at a fixed time-point after CRT (e.g., at 1- or 2-months post-CRT) and these series were labeled ‘time-point’ studies for the purpose of our analysis (Table 1). Another four studies tabulated the incidence of lymphopenia as *any* occurrence of lymphopenia from the start of CRT to some defined time (e.g., end of CRT, 1-month post-CRT, or 2-month post-CRT, etc.). These series were labeled ‘time-range’ studies (Table 1). One study (Byun et al.) reported and analyzed lymphopenia separately both as a ‘time-point’ and ‘time-range’ [15]. The overall incidence of lymphopenia for the studies ranged from 2.9 to 46.6%, with a combined average of 31.8% (utilizing the criteria of lymphopenia defined by each individual study in terms of timing and cutoff value). The average incidence of lymphopenia differed between time-point and time-range studies, measuring 11.8% and 39.9%, respectively. For each of these studies, all but one (Mohan et al.) demonstrated a statistically significant lower median OS associated with lymphopenia patients versus non-lymphopenia patients (Table 1) [19].

**Fig. 2:**
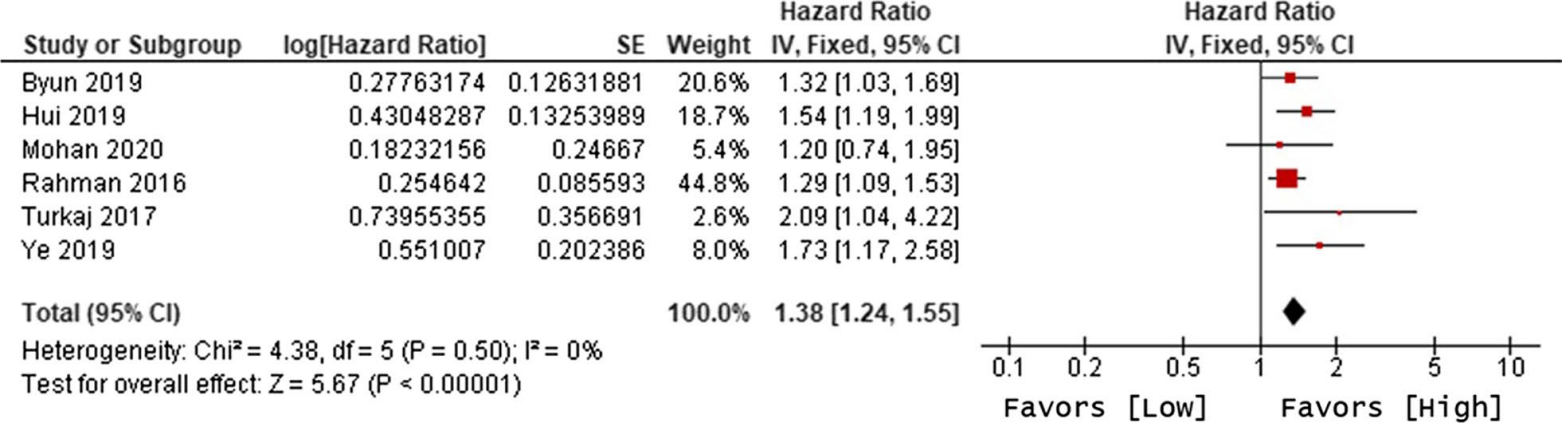
Forest plot for lymphopenia and overall survival of time-point studies

**Fig. 3:**
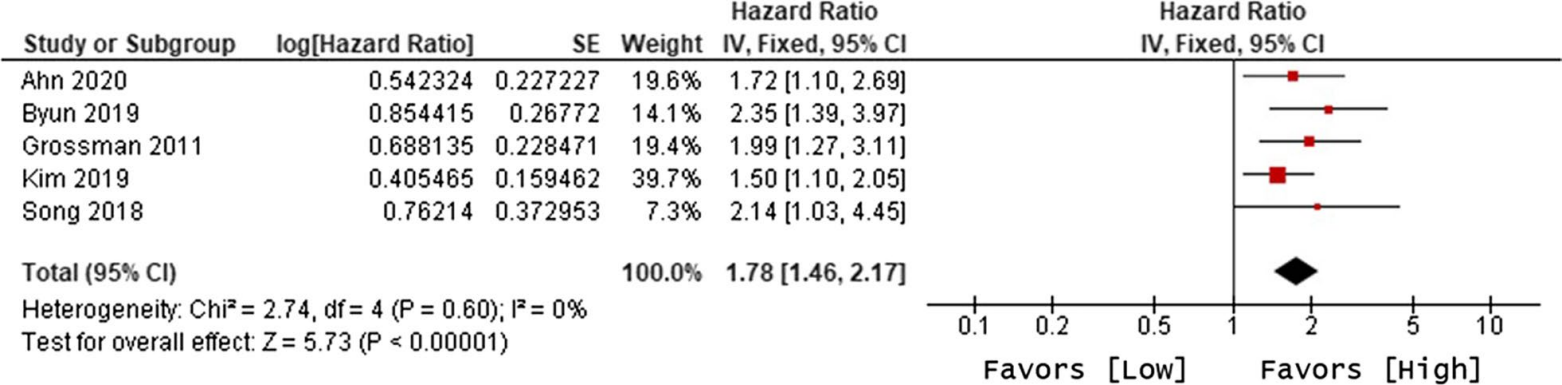
Forest plot for lymphopenia and overall survival of time-range studies

The primary objective of our analysis was to estimate the magnitude of association between lymphopenia development and OS in the published literature. Given the inherit different nature by which lymphopenia was defined between time-point and time-range studies, the analysis was dichotomized with separate pooled HR analysis performed for the time-point and time-range studies. Lymphopenia was associated with increased risk of death for both study types, with a pooled HR of 1.78 (95% CI 1.46–2.17, P < 0.00001) for the time-range studies, and a pooled HR of 1.38 (95% CI 1.24–1.55, P < 0.00001) for the time-point studies (Figs. 2 and 3). There was minimal overall HR heterogeneity among the studies (either for time-point or time-range) with I^2^ = 0% for both.

## Discussion

Given the increasingly recognized link between treatment-related lymphopenia and poor survival outcomes for GBM patients, we performed a systematic literature review and pooled analysis to better quantify this association. After identifying 10 studies that met our inclusion criteria, our pooled analysis confirms that GBM patients who experience lymphopenia have an inferior OS with a pooled HR of 1.78 for the time-range studies and a HR of 1.38 for the time-point studies. This significant association highlights the importance of the immune system and lymphocytes in particular for the survival of GBM patients.

Lymphocytes play a crucial role in host defense against pathogens and elimination of tumor cells. The latter mechanism may be responsible for the inferior survival of GBM patients. Grossman et al.’s seminal study was the only study that examined cause of death and demonstrated that lymphopenic patients did not develop higher rates of infection and their cause of death was almost entirely due to early tumor progression [11]. Lymphopenia is particularly important for GBM given it is classified to be an immunologically ‘cold’ tumor due to its poor response to immunotherapy [22–24]. Mechanistically, this is thought to be due to its immune privileged site of origin, low antigen burden, and inhibitory tumor microenvironment [25, 26]. As highlighted by our review and analysis, another possible contributor to poor immunotherapy response may be iatrogenic lymphopenia from the current standard-of-care treatments (i.e., TMZ and RT). Checkmate-548 failed to show a benefit to the addition of nivolumab (compared to placebo) to standard TMZ + RT for newly diagnosed GBM patients [22]. The lack of efficacy may in part be due to impairment and depletion of lymphocytes, whose activity is critical to the efficacy of Nivolumab.

Efforts to mitigate treatment-related lymphopenia may improve outcomes of GBM patients. From a radiation therapy standpoint this can be accomplished by hypofractionation, reducing treatment volumes, and possibly with the use of proton therapy. Regarding reduced treatment volumes, Rudra et al. examined the impact of limiting the radiation treatment volume from targeting the MRI T2 abnormality (the standard United States volume) to targeting just the T1 contrast enhancement [27]. The T1-based planning treatment volumes (PTVs) were smaller compared to the T2-based PTVs (375 cc vs. 245.7 cc, p < 0.001), and interestingly patients treated with the smaller T2-based PTVs had a trend toward decreased lymphopenia at 3-months after the start of CRT (15.5% vs. 33.8%, p = 0.12). Their analysis also uncovered brain V25 Gy as independent predictor of developing lymphopenia, highlighting the importance of sparing radiation to uninvolved brain, which presumably spares dose to circulating lymphocytes. Another means of limiting radiation to the brain is with the use of proton therapy, due to its inherent property of lacking an exit dose. Mohan et al. examined lymphopenia (a study included in our pooled analysis) in patients on a randomized prospective phase II trial comparing proton therapy and conventional photon-based RT [19]. Their analysis found that patients undergoing proton therapy (compared with photon-based RT) had lower whole brain V25 Gy (35.3 cc vs. 43.8 cc) and had lower rates of developing lymphopenia (15% vs. 39%, P = 0.024).

Other means of mitigating lymphopenia involve directly boosting lymphocyte counts via lymphocyte re-infusion, recombinant IL-7, and transient sequestration of lymphocytes using fingolimod. Early-stage trials testing these interventions for GBM patients are already underway with the intention to improve survival outcomes [28–30].

Another important possible treatment-related lymphopenia contributor (other than TMZ and RT) for GBM patients is corticosteroids, which are commonly used for GBM patients to control vasogenic brain edema and associated symptoms. Corticosteroids have a well-documented lymphotoxic effect and cause lymphopenia [31, 32]. One of our included studies, Hui et al., analyzed the impact of corticosteroid use on survival outcomes of GBM patients [16]. Results demonstrated that patients who received higher doses of corticosteroids (> 2 mg/day) had significantly higher rates of developing lymphopenia and also had decreased OS. In a meta-analysis of 22 studies by Petrelli et al., steroid use in GBM patients was also associated with decreased OS [33]. The interpretation of corticosteroid-inducted lymphopenia association with decreased OS is challenging given the known morbidity of additional corticosteroid-induced side effects (hyperglycemia, hypertension, electrolyte abnormalities, etc.). Additionally confounding the association between corticosteroid use and OS is the fact that steroids are typically only used in symptomatic patients, thus corticosteroids may be functioning as a marker of larger tumor burden and/or progressing disease.

Our review of the literature uncovered a lack of uniformity in defining lymphopenia across the various studies in terms of the cutoff value and the timing of when lymphopenia is recorded as an event. The cutoff values ranged from 400 to 1000 TLC per cc, though the majority of studies used a cutoff of < 500 TLC per cc, which is the CTCAE grade 3 lymphopenia classification [34]. This could bias the effect size estimates if the cutoff was chosen to maximize the contrast between patients with and without lymphopenia. Clearly, this bias would be inherited in our meta-analysis. Regarding the timing of lymphopenia, about half of the included studies defined lymphopenia at a time-point and the other half over a time-range. Though our separate analyses of the time-point and time-range studies both demonstrated a significant association between lymphopenia and OS, differences in these definitions should be noted. There is a greater likelihood of classifying a patient as lymphopenic with the time-range definition (compared to time-point definition) since this captures any lymphopenia event over typically a 3-month window (from start of CRT to 1-month post-CRT, for instance). In contrast, there is presumably a lesser likelihood of classifying a patient as lymphopenic using the specific time-point definition (e.g., at 1-month post-CRT) given the singularity of the allowable time. Thus, the time-range definition may inflate the incidence of lymphopenia, which is supported by our analysis demonstrating a higher incidence of lymphopenia for time-range studies when compared with time-point studies (about 40% vs. 12%, respectively). Also, the time-point versus time-range may have differing biological implications. The time-range criteria may include patients who experience lymphopenia early in the course of CRT, but ultimately recover some time after. However, the time-point definition captures patients who may have experienced lymphopenia at some point during CRT, but fail to recover or who experience persistent lymphopenia. The persistence of lymphopenia may lead to worse tumor control and other sequalae. Interestingly, Byun et al. was the only study to conduct analysis using both time-point and time-range studies [15]. They found that using either definition, lymphopenia was significantly associated with worse OS on univariate analysis; however, only the time-point definition showed significance on multivariate analysis.

Limitations of our analysis should be noted. First, the majority of the included studies were retrospective single-institution experiences, with the exception of Grossman et al. (a multicenter prospective study) and Mohan et al. (a prospective single-instruction randomized phase II clinical trial) [11, 19]. There are inherent biases to such single-institution retrospective studies which can introduce confounding factors such as inclusion of varied patient populations (e.g., inclusion of grade III gliomas, low performance status patients, unknown MGMT status), differences in treatment (e.g., RT above and below standard 60 Gy). Second, with the exception of Mohan et al., all of the included studies were positive (i.e., found that lymphopenia correlated with worse survival) [19]. This naturally raises the possibility of positive publication bias, where analyses that did not demonstrate a correlation between treatment-related lymphopenia and survival were not published. Of note, Mohan et al. was one of the only studies in our pooled analysis that did not show lymphopenia associated with worse OS [19]. Notably, this was also the study with the fewest patients (N = 84) which affected the power to detect a given effect size. Third, given this was a pooled analysis from existing literature, patient-level data was not available and therefore patient/treatment factors could not be controlled. Further, even though individual studies created adjusted hazard ratios accounting for covariates, given each study used different prognostic factors in these models, we could not construct pooled adjusted model of the hazard ratios.

These limitations highlight the need for prospectively collected data from large well defined patient populations homogenously treated and with a predefined statistical analysis plan for testing the prognostic effect of lymphopenia. As an example of potential differences between the retrospective studies and prospective RCT data, RTOG 0825’s standard arm of patients (N = 300) undergoing standard CRT (experimental arm was standard CRT + bevacizumab) had an 7.3% incidence of lymphopenia (defined as TLC < 500 per cc, over the time-range of CRT), which is much lower than the average incidence of 40% for the time-range studies included in our analysis [35]. Encouragingly, lymphopenia is a pre-specified exploratory endpoint in the open NRG BN-001 radiation dose-escalation trial which is also evaluating outcomes in patients treated with protons vs. photons [36].

## Conclusion

This pooled analysis shows a significant association between treatment-related lymphopenia and decreased OS in GBM patients undergoing CRT, consistent with the majority of the published studies. Future and ongoing prospective data will help confirm these findings. From a clinical standpoint, efforts to minimize lymphopenia (when feasible) would be prudent but it remains to be clarified if such measures will lead to improved outcomes for GBM patients. Further, in the expanding era of immunotherapy, it remains to be tested whether limiting or reversing lymphopenia (e.g., lymphocyte re-infusion, lymphocyte expansion with IL-7) may help uncover the yet to be realized potential of immunotherapy for the treatment of GBM.

## Data Availability

The datasets supporting the conclusions of this article are included within the article.

## Abbreviations

ASCO: American Society of Clinical Oncology
ASTRO: American Society for Radiation Oncology
CI: Confidence interval
CRT: Chemo-radiation therapy
CTV: Clinical treatment volume
CTCAE: Common terminology criteria for adverse events
GBM: Glioblastoma multiforme
HGG: High grade glioma
HR: Hazard ratio
MGMT: O6-methylguanine-DNA-methyltransferase
MRI: Magnetic resonance imaging
OS: Overall Survival
PRIMSA: Preferred Reporting Items for Systematic Reviews and Meta-Analyses
PTV: Planning treatment volume
RCT: Randomized control trial
RT: Radiation therapy
RTOG: Radiation therapy oncology group
SNO: Society of Neuro-Oncology
TLC: Total lymphocyte count
TMZ: Temozolomide
