# Correction: Systematic review and pooled analysis of the impact of treatment-induced lymphopenia on survival of glioblastoma patients

**DOI:** 10.1186/s13014-024-02443-4

**Published:** 2024-05-15

**Authors:** Ali M. Saeed, Søren M. Bentzen, Haroon Ahmad, Lily Pham, Graeme F. Woodworth, Mark V. Mishra

**Affiliations:** 1 Department of Radiation Oncology, Greenebaum Comprehensive Cancer Center, University of Maryland, University of Maryland School of Medicine, Baltimore, USA; 2 Maryland Proton Treatment Center, Baltimore, MD USA; 3 Department of Epidemiology and Public Health, Division of Biostatistics and Bioinformatics, University of Maryland School of Medicine, Baltimore, USA; 4 Department of Medical Oncology, Greenebaum Comprehensive Cancer Center, University of Maryland, University of Maryland School of Medicine, Baltimore, USA; 5 Department of Neurosurgery, University of Maryland School of Medicine, Baltimore, MD USA

**Radiation Oncology (2024) 19:36**

10.1186/s13014-023-02393-3

After publication of this article, the authors reported that the author names ‘Ali M. Saeed, Søren M. Bentzen, Haroon Ahmad, Lily Pham, Graeme F. Woodworth and Mark V. Mishra’ were incorrectly written as ‘A. M. Saeed, S. M. Bentzen, H. Ahmad, L. Pham, G. F. Woodworth and M. V. Mishra’.

The original article has been updated by the authors.

